# The use of oral dutasteride, minoxidil, and finasteride for treating endocrine therapy-induced hair loss from breast cancer treatment: A case series study

**DOI:** 10.1016/j.jdcr.2025.11.001

**Published:** 2025-11-07

**Authors:** Abigail Katz, Celina Dubin, Katrina April David, Benjamin Ungar, Nicholas Gulati, Angela J. Lamb

**Affiliations:** Department of Dermatology, Icahn School of Medicine at Mount Sinai, New York, New York

**Keywords:** breast cancer aromatase therapy, endocrine therapy-induced alopecia, hair loss, oral dutasteride, oral finasteride, oral minoxidil

## Introduction

Breast cancer (BrCa) is the most frequently diagnosed cancer among women in the United States, with approximately 310,720 new cases annually.^1^^,^^2^ Treatment regimens vary by BrCa type and hormone receptor status, and may include a combination of surgery, chemotherapy, radiation, and endocrine therapy (ET) with aromatase inhibitors (AIs) or selective estrogen receptor modulators (SERMs).^1^^,^^2^ ET is considered the standard of care maintenance treatment for hormone receptor positive (HR+) BrCa, which accounts for 70% to 80% of all cases.^1^

AIs and SERMs prevent tumor growth and recurrence by suppressing estrogen levels through estrogen receptor blockade and inhibiting the conversion of androgens to estrogens.^3^^,^^4^ To be effective, ET requires daily treatment for up to 10 years after diagnosis.^2^^,^^3^ Though generally well tolerated, 15% to 33% of patients on ET develop endocrine therapy-induced alopecia (EIA), which characteristically presents with diffuse thinning over the frontal and vertex scalp from ET induced hormone changes.^2^^,^^5^^,^^6^ In addition to scalp involvement, some patients anecdotally report eyebrow thinning, though this has not been well studied.^6^ The resulting changes in hair density can be detrimental to self-esteem and quality of life.^2^^,^^5^ One study reported that up to 8% of patients elected to discontinue ET specifically because of hair loss.^4^^,^^7^

Treatment options for EIA are limited and there are no FDA approved options. The pathogenesis of EIA involves hormonal imbalances in the hair follicle, closely resembling the hair loss mechanism in androgenic alopecia.^3^ At the hair follicle, estrogen plays a protective role by promoting and maintaining the anagen (growth) phase of the hair cycle, while dihydrotestosterone (DHT) causes miniaturization of the follicle and hair shedding, the root cause of androgenic alopecia.^6^^,^^8^ Endocrine therapy similarly results in an increased androgen to estrogen ratio at the follicle, either by directly preventing the conversion of androgens to estrogens with AIs, or by directly blocking the estrogen receptor and preventing binding with SERMs.^5^^,^^6^ Importantly, this hormonally mediated hair loss in EIA differs from chemotherapy-induced hair loss, which is caused by inhibition of the proliferating matrix keratinocyte cells of the hair bulb, leading to apoptosis and temporary shedding of the hair follicle.^2^^,^^3^

Androgenic alopecia has been successfully managed with oral agents including minoxidil, which causes vasodilation and enhances follicular blood flow and nutrient delivery, as well as finasteride and dutasteride, which are both 5-alpha-reductase inhibitors that suppress DHT levels and mitigate the androgen-mediated follicle miniaturization.^4^^,^^6^^,^^8^ Given the shared mechanism of hair loss, treatments that have shown promising results for androgenetic alopecia may also be advantageous in EIA.^3^^,^^9^ One case report demonstrated that 3 months of daily oral dutasteride with topical minoxidil 5% increased hair density in 70-year-old woman on tamoxifen therapy.^4^ Another study found that topical plus oral minoxidil was associated with increased hair regrowth in EIA patients compared to topical minoxidil alone.^10^

The management of EIA with hormone-modifying medications poses a unique clinical challenge in this patient cohort because their oncologic disease is also hormonally mediated. While oral minoxidil offers a non-hormonal option, the use of 5-alpha-reductase inhibitors warrants consideration. In the majority of patients, finasteride and dutasteride have not been associated with consistent increases in circulating estrogen, though elevations have been reported in a subset of women.^3^^,^^4^^,^^11^ There is also limited data on the use of these medications in breast cancer survivors specifically and, the risk of developing breast cancer with 5-α-reductase inhibitors has not been well established, though current data suggests a low risk.^12^^,^^13^

The present retrospective case series explores the utility of oral hair loss medications for the treatment of EIA. Eight patients with EIA treated with either oral minoxidil, finasteride, and/or dutasteride for at least 3 months were included. All patients had completed active cancer therapy and were regularly monitored by their oncologists. Pretreatment and posttreatment photographs were compared by 2 independent dermatologists using a standardized global photographic assessment (GPA), a 7-point scale (−3 to +3) to quantify change in hair density.^14^ The goal of this study was to assess hair growth outcomes in a small retrospective cohort of EIA patients treated with an oral medication and gather preliminary evidence about their potential use in breast cancer survivors. Due to the retrospective design, this study could not adequately assess safety and tolerability of these medications and further research will be needed given the hormonally sensitive nature of most breast cancers.

## Patient cases

A comprehensive summary of all patient results is presented in Table I.

**Table I:** Summary of patient characteristics, treatments, and GPA ratings

Patient	Age	Breast cancer subtype	Endocrine therapy/ies (duration months) (all once daily)	Hair loss medication and dose (all once daily)	Hair loss treatment duration (m)	Average GPA∗	Side effects
1	71	ER+/PR+/HER2-	Anastrozole1 mg (9 m)	Minoxidil 1.25 mg	10	+0.5	None reported
			Exemestane 25 mg (15 m)				
			Letrozole 2.5 mg (19 m)				
2	80	ER+/PR+/HER2-	Anastrozole 1 mg (6 m)	Minoxidil 1.25 mg	9	+1	Facial hirsutism
3	48	ER+/PR+/HER2-	Exemestane 25 mg (8 m)	Minoxidil 1.25 mg	16	+2	Facial hirsutism
4	45	ER+/PR+/HER2+	Tamoxifen 20 mg (7 m)	Minoxidil 1.25 mg	3.5	+2	Facial hirsutism
5	67	ER+/PR+/HER2-	Tamoxifen 20 mg (36 m)	Dutasteride 0.5 mg	13	+1	None reported
6	57	ER+/PR+/HER2-	Tamoxifen 20 mg (48 m)	Dutasteride 0.5 mg	15	+1.5	None reported
			Anastrozole 1 mg (5 m)				
			Exemestane 25 mg (18 m)				
7	72	ER+/PR-/HER2-	Anastrozole 1 mg (16 m)	1st: dutasteride 0.5 mg + topical minoxidil 5% 2nd: minoxidil 2.5 mg + dutasteride 0.5 mg + topical minoxidil 5%	18 (dutasteride) 19 (dutasteride + minoxidil)	+0.5 (dutasteride) +1.5 (dutasteride + minoxidil)	None reported
			Tamoxifen 20 mg (6 m)				
8	76	ER+/PR+/HER2-	Anastrozole 1 mg (not specified)	Finasteride 2.5 mg	60	+2.5	None reported
				+ topical minoxidil 5%			

∗ Global Photographic Assessment (GPA) is a standardized assessment to measure the change in hair density between 2 images of the scalp. It uses a 7-point grading scale, (ranging from −3 to + 3), to quantify the change in density with measurements as follows: −3 (severely decreased), −2 (moderately decreased), −1 (mildly decreased), 0 (no change), +1 (mildly increased), +2 (moderately increased), +3 (greatly increased).^14^

### Minoxidil

Four female patients ages 45 to 80 years old with EIA received oral minoxidil 1.25 mg daily. ET treatment regimen and duration of ET prior to establishing care for hair loss varied between patients, and included anastrozole 1 mg daily for 6 months, exemestane 25 mg daily for 8 months, tamoxifen 20 mg daily for 7 months, and letrozole 2.5 mg daily for 19 months.

**Fig 1:**
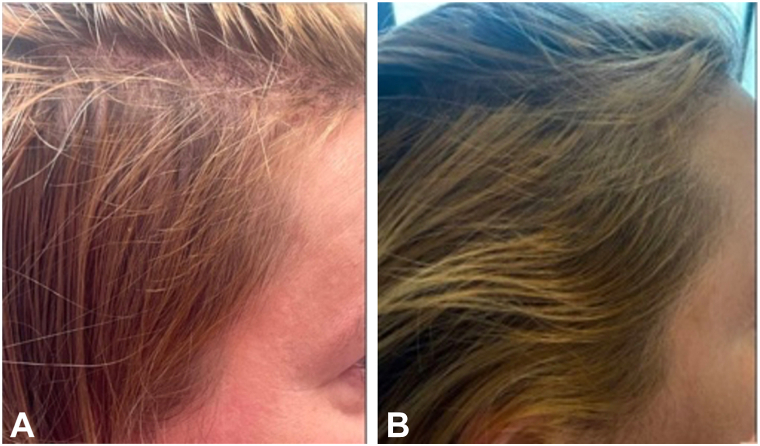
Minoxidil. Patient before **(A)** and 3.5 months after **(B)** oral minoxidil 1.25 mg daily.

All 4 patients presented to dermatology clinic with a chief complaint of hair loss and excess shedding, with physical exams notable for decreased areas of hair density in the frontotemporal and vertex areas of the scalp. All 4 patients were treated with oral minoxidil 1.25 mg daily for an average of 9 months (range 3.5-16 months). Patient self-reported outcomes included both moderate and significant hair regrowth, and 1 patient reported primarily decreased shedding. The average GPA for the change in hair density as determined by 2 independent dermatologist reviewers was +1.3 (range + 0.5 to +2), equating to overall “mild to moderate” regrowth (Fig 1).

Minoxidil therapy was overall well-tolerated without any reports of irritation or discomfort. The only notable side effect was facial hirsutism, which was reported by 3 of the 4 patients. All 3 of the patients with hirsutism continued treatment with oral minoxidil despite experiencing this side effect.

Three female patients ages 57 to 72 years old with EIA were treated with oral dutasteride 0.5 mg daily. All 3 patients had taken tamoxifen for a period of 6, 36, and 48 months, respectively. Two patients had also taken anastrozole, 1 for 16 months and another for 5 months, followed by exemestane for 18 months.

**Fig 2:**
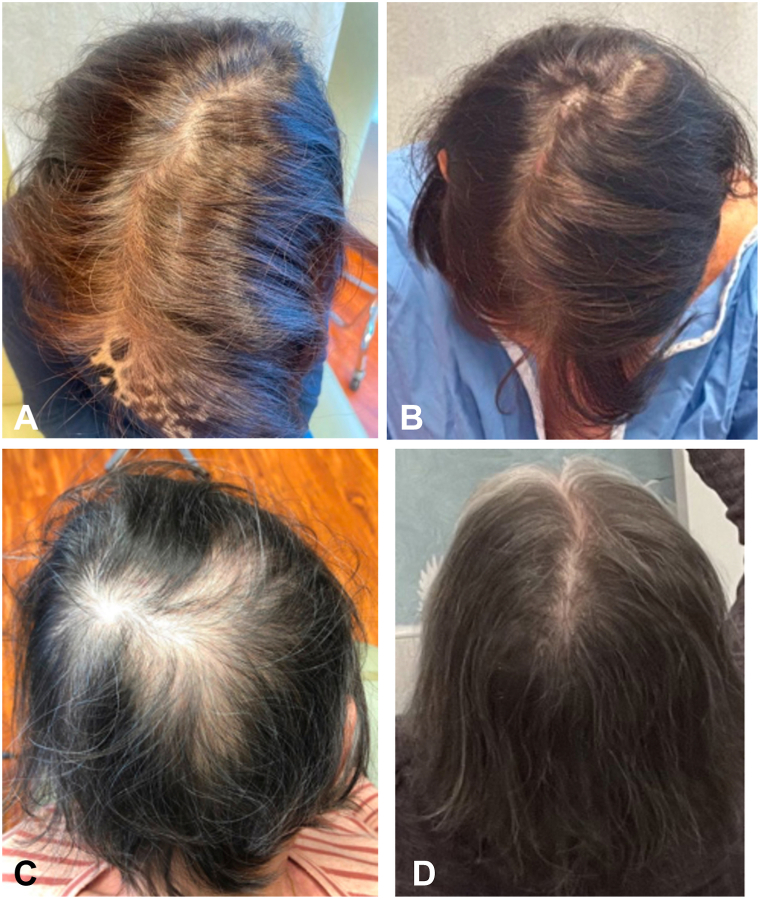
Dutasteride A patient before **(A)** and 15 months after **(B)** oral dutasteride 0.5 mg daily. A second patient before **(C)** and 37 months after **(D)** oral dutasteride 0.5 mg daily, oral minoxidil 2.5 mg daily, and topical minoxidil 5% daily.

Upon presentation to dermatology clinic, all 3 patients had decreased hair density in the frontotemporal and vertex regions of the scalp. Three patients were treated with oral dutasteride 0.5 mg daily for an average of 15 months (range 13-18 months). One patient also used topical minoxidil 5% once daily. All patients self-reported decreased hair shedding and increased regrowth over this time. The average GPA as rated by 2 independent dermatologists was +1 (range = +0.5 to +1.5), equating to “mild” regrowth (Fig 2, *A* and *B*). Treatments were extremely well tolerated and there were no reported side effects.

One patient who took oral dutasteride 0.5 mg daily in conjunction with topical minoxidil 5% daily achieved an initial 10% improvement in hair density on physical exam, with an average GPA of +0.5. However, after 18 months of treatment, she reported symptom stabilization without any further regrowth. Given lack of continued progress, oral minoxidil 2.5 mg daily (started as 1.25 mg daily for 2 weeks before increasing to 2.5 mg) was added to her regimen alongside dutasteride and topical minoxidil. Eighteen months later, the patient reported moderate hair and eyebrow regrowth, also evident on physical exam. Average GPA after the addition of oral minoxidil was +1.5, equating to “mild to moderate” regrowth (Fig 2, *C* and *D*).

**Fig 3:**
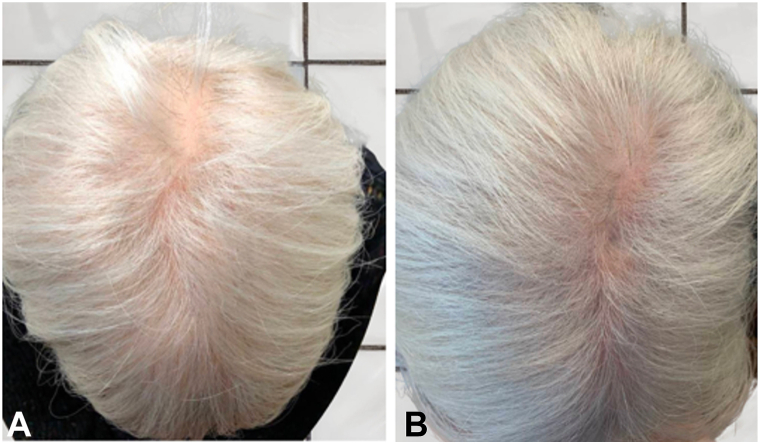
Finasteride A patient before **(A)** and 16.5 months after **(B)** oral finasteride 2.5 mg daily and topical minoxidil 5% daily.

One 75-year-old with EIA from anastrozole took 2.5 mg finasteride daily and topical minoxidil 5% (used intermittently) for a duration of 60 months. The patient self-reported slight increases in hair density over time, also observed on exam and she continues to be stable on this regimen. There were no reported side effects. Average GPA was +2.5, “moderate to significant” regrowth (Fig 3).

## Discussion

This case series evaluated oral minoxidil, dutasteride, and finasteride for the treatment of EIA in women on breast cancer therapy. All 8 patients demonstrated at least some degree of hair regrowth and stabilization of shedding during treatment. The amount of regrowth, however, varied between patients, with some showing “slight-to-no” change in hair density while others demonstrated “moderate-to-significant” improvement. The variation in treatment response is likely multifactorial. The patient ages ranged from 45-80 (average 64.5 years), the duration of ET treatment prior to establishing dermatologic evaluation for hair loss ranged from 6 to 36 months (average 13 months), and the treatment time on an oral hair loss medication ranged from 4-60 months (average 11 months). In addition, the specific type of ET varied between patients, with the most common being tamoxifen, anastrozole, or exemestane. These differences, in addition to patient genetics, breast cancer subtype and treatment modalities, environmental factors, patient compliance, and others, may contribute to the range of patient response.

When comparing oral EIA medications, the average GPA change in hair density was slightly higher among patients taking oral minoxidil alone (GPA +1.3) compared to dutasteride alone (GPA +1). However, the average response to both oral minoxidil and dutasteride would be categorized as a “slight” improvement in hair density and the true difference in efficacy cannot be determined from this case series alone. The additional hair regrowth observed in 1 patient after oral minoxidil was added to the regimen of oral dutasteride and topical minoxidil suggests that these medications may work synergistically to support hair growth via multiple mechanisms. The patient on combination therapy was also noted to have eyebrow regrowth which may represent a novel observation that requires further investigation. Across all patients, the greatest degree of hair regrowth was “moderate” in the patient on finasteride plus topical minoxidil 5%. Future research should examine the possible additive benefits of combining multiple medications for EIA treatment and clarify which regimen yields the maximum regrowth potential.

All 3 oral hair loss medications appeared to be well tolerated without any reported significant adverse reactions while on treatment. The only documented side effect was facial hirsutism in 3 of 4 patients taking oral minoxidil, which is a commonly observed side effect.^14^^,^^15^ The long-term use of hormone-modifying medications in breast cancer survivors on endocrine therapy raises important safety considerations. These agents modulate hormonal pathways, and a subset of patients may experience a small increase in estrogen, though the clinical significance of this is unknown. While it is not clinically necessary to check estrogen labs before starting EIA medications, little is known about their impact on hormone levels in breast cancer survivors and further research in this area is critical. At this time, there is no evidence that finasteride increases breast cancer risk, however, the literature is limited and controlled studies are needed.^11^^,^^13^ In the present case series, all patients had completed active cancer treatment, were hormonally postmenopausal as a result of therapy, and remained under routine cancer surveillance with their oncologists. No adverse oncologic or hormone-related events were noted during treatment; however, the short time period would likely not be sufficient to adequately assess long-term risk. Oral hair loss medications such as 5-alpha-reductase may be an efficacious treatment option for EIA however, their safety profile and tolerability in this patient population have yet to be established.

Oral minoxidil, dutasteride, and finasteride all promoted hair regrowth and may offer promising solutions for select patients suffering from EIA. Prospective studies and clinical trials are warranted to investigate the true potential of these medications, both individually and possibly in combination, for the treatment of EIA to improve options for patients suffering from this commonly occurring side effect of breast cancer therapy.

## Conflict of interest

None disclosed.
